# Early-life exposures and age at thelarche in the Sister Study cohort

**DOI:** 10.1186/s13058-021-01490-z

**Published:** 2021-12-11

**Authors:** Mandy Goldberg, Aimee A. D’Aloisio, Katie M. O’Brien, Shanshan Zhao, Dale P. Sandler

**Affiliations:** 1 Epidemiology Branch, National Institute of Environmental Health Sciences, 111 TW Alexander Dr., Research Triangle Park, NC 27709 USA; 2 Social and Scientific Systems, Durham, NC USA; 3 Biostatistics and Computational Biology Branch, National Institute of Environmental Health Sciences, Research Triangle Park, NC USA

**Keywords:** Puberty, Breast development, Thelarche, Menarche, Early-life, Prenatal exposures

## Abstract

**Background:**

Early age at breast development (thelarche) has been associated with increased breast cancer risk. Average age at thelarche has declined over time, but there are few established risk factors for early thelarche. We examined associations between pre- and postnatal exposures and age at thelarche in a US cohort of women born between 1928 and 1974.

**Methods:**

Breast cancer-free women ages 35–74 years who had a sister diagnosed with breast cancer were enrolled in the Sister Study from 2003 to 2009 (N = 50,884). At enrollment, participants reported information on early-life exposures and age at thelarche, which we categorized as early (≤ 10 years), average (11–13 years), and late (≥ 14 years). For each exposure, we estimated odds ratios (ORs) and 95% confidence intervals (CIs) for early and late thelarche using polytomous logistic regression, adjusted for birth cohort, race/ethnicity and family income level in childhood.

**Results:**

Early thelarche was associated with multiple prenatal exposures: gestational hypertensive disorder (OR = 1.25, 95% CI 1.09–1.43), diethylstilbestrol use (OR = 1.23, 95% CI 1.04–1.45), smoking during pregnancy (OR = 1.20, 95% CI 1.13–1.27), young maternal age (OR 1.30, 95% CI 1.16–1.47 for < 20 vs. 25–29 years), and being firstborn (OR = 1.25, 95% CI 1.17–1.33). Birthweight < 2500 g and soy formula use in infancy were positively associated with both early and late thelarche.

**Conclusions:**

Associations between pre- and postnatal exposures and age at thelarche suggest that the early-life environment influences breast development and therefore may also affect breast cancer risk by altering the timing of pubertal breast development.

**Supplementary Information:**

The online version contains supplementary material available at 10.1186/s13058-021-01490-z.

## Background

While age at menarche is an established breast cancer risk factor [1], earlier age at onset of breast development (thelarche) has also recently been linked to increased breast cancer risk, independent of age at menarche [2, 3]. Identifying modifiable risk factors associated with early thelarche may provide an opportunity for primary prevention of breast cancer by delaying the onset of pubertal breast development. Apart from larger childhood body size [4, 5], however, there are few established risk factors for early thelarche.

A recent meta-analysis estimated that age at thelarche has declined at a rate of 3-months per decade over the past 50 years [6]. This rapid rate of decline suggests that environmental factors, acting independently or interacting with genetic susceptibility, are driving the secular trend [7]. Factors hypothesized to influence the timing of thelarche include nutritional factors, psychosocial stressors, and exposure to endocrine-disrupting chemicals, as well as conditions affecting the intrauterine environment [6–8].

Our objective was to examine associations between pre- and postnatal exposures and age at thelarche in a prospective US cohort of women born between 1928 and 1974. We further examined whether associations between early-life exposures and age at thelarche were modified by other factors associated with early thelarche, including being born in a more recent birth cohort [6], African-American/Black or Hispanic/Latina identity [4], lower socioeconomic status [9], and increased familial risk of breast cancer [10].

## Methods

### Study population

The Sister Study is a prospective cohort designed to investigate environmental and genetic risk factors for breast cancer (for more details, see [11]). From 2003 to 2009, 50,884 women enrolled in the cohort. Women were eligible if they lived in the USA including Puerto Rico, were between the ages of 35–74 years, and had a sister diagnosed with breast cancer, but were breast cancer-free themselves at enrollment.

Women completed a computer-assisted telephone interview at baseline which included information on demographics, reproductive and lifestyle factors, and medical and family history. Women also completed a self-administered family history questionnaire that included questions about potential maternal, in utero and infancy exposures. We utilized baseline data from Sister Study Data Release 7.2 for this analysis.

All participants provided written informed consent. The institutional review board of the National Institutes of Health approved the study.

### Pubertal timing assessment

At the baseline interview, women reported the age in years when they first noticed their breasts developing. Alternatively, women reported their grade in school, which we converted to age (1.2% of the cohort reported grade only). We excluded women who reported that thelarche occurred at age 21 or older, which we considered to be implausible. We categorized timing of thelarche as early (≤ 10 years), average (11–13 years) and late (≥ 14 years) based on the distribution of age at thelarche in the cohort (Additional file 1: Fig. S1). Women also reported their age at menarche, which we categorized as early (≤ 11 years), average (12–13 years) and late (≥ 14 years).

### Early-life exposure assessment

We considered participants’ perinatal environment, which included what they may have been exposed to while in utero or during infancy. In utero exposures included mothers’ exposures to pre-pregnancy and pregnancy-related diabetes, pregnancy-related hypertension, pre-eclampsia, eclampsia or toxemia, diethylstilbestrol (DES) use during pregnancy, living or working on a farm during pregnancy, and smoking during pregnancy using four response categories. For all of these, we considered responses of “definitely” and “probably” as exposed and categorized “probably not” and “definitely not” as unexposed. We defined gestational diabetes as a report of pregnancy-related diabetes and no report of pre-pregnancy diabetes, and gestational hypertension as a report of pregnancy-related hypertension and no report of pre-eclampsia, eclampsia, or toxemia. We did not collect information on type of pre-pregnancy diabetes or on pre-pregnancy hypertension. We also considered any diabetes and any gestational hypertensive disorder. Maternal age at the participant’s birth was reported continuously, with categorical options provided if the participant did not know the exact age.

Additional birth- and infancy-related exposures we considered were birthweight, gestational age, multiple gestation, birth order, and type of infant feeding. Participants reported their own birthweight in pounds and ounces. If unknown, they were asked if they weighed more or less than 5lbs at birth. We converted birthweight to grams and categorized it into clinically relevant categories (< 2500 g, 2500–3999 g and ≥ 4000 g). Participants reported whether they were born within one week of their mothers’ due date, and if not, whether they were born less than 2 weeks, 2–4 weeks, 1–2 months, or more than 2 months before or after. We categorized gestational age at birth as born ≥ 1 month before, 2–4 weeks before, or not born ≥ 2 weeks before the due date. Participants reported if they were part of a multiple birth (including stillbirths). We classified participants as firstborn or not based on the birth dates of full siblings and maternal half-siblings ascertained in baseline questionnaires. Participants reported if they were ever breastfed and if they were ever fed soy formula as an infant separately using four response categories, which we dichotomized as described for maternal exposures.

### Covariate assessment

We categorized birth year into approximately 10-year intervals (1928–1939, 1940–1949, 1950–1959 and 1960–1974). Women self-identified their race as American Indian or Alaska Native, Asian, Black or African-American, Native Hawaiian or other Pacific Islander, and/or White. They also reported if they considered their ethnicity to be Hispanic or Latina. We categorized race/ethnicity as non-Hispanic White, non-Hispanic African-American/Black, Hispanic/Latina, and others, which included women who identified as Asian/Pacific Islander, American Indian/Alaska native, or who did not specify race, and did not identify as Hispanic/Latina. Qualitative family income level growing up (well-off, middle income, low income, or poor) and relative weight at age 10 (heavier, same weight as, or lighter than peers) were both reported at enrollment. We also collected detailed information on breast cancer family history, which we used to calculate a continuous Bayesian family history score (BFHS) to assess familial risk. This score was developed in the Sister Study cohort and incorporates family size, number and age at diagnosis of breast cancer cases in first-degree relatives, and current age or age at death for non-cases (for more details, see [12, 13]).

### Analytic sample

Of the 50,884 women enrolled in the cohort, we excluded 3 women who withdrew their data and 810 women who did not complete the family history questionnaire (Additional file 2: Fig. S2). Since we were interested in in utero exposures, we also excluded women who reported that they were adopted (n = 188). We excluded 609 women with missing age at thelarche (n = 576) or thelarche reported at age 21 or older (n = 33). Lastly, we excluded women with missing data on race/ethnicity and/or childhood family income (n = 112). This left an analytic sample of 49,162 women.

### Statistical analysis

We examined the distributions of demographic and early-life factors by timing of thelarche. We used polytomous logistic regression to estimate odds ratios (ORs) and 95% confidence intervals (CIs) for associations of early-life exposures with early (≤ 10) and late thelarche (≥ 14) relative to average age (11–13 years). We adjusted for birth cohort, race/ethnicity, and childhood family income. We additionally adjusted for relative weight at age 10 to examine whether associations were independent of childhood body size, but we did not conduct a formal mediation analysis due to ambiguity about the relative timing since thelarche ranged from 4 to 20 years. We excluded 129 women with missing data for relative weight at age 10 from these analyses.

We examined whether associations between early-life exposures and timing of thelarche were modified by birth cohort, race/ethnicity, childhood family income and relative weight at age 10 through stratification and tested for statistical heterogeneity using a likelihood ratio test. We estimated stratum-specific associations for non-Hispanic White, non-Hispanic African-American/Black and Hispanic/Latina women only. We examined effect modification by extent of breast cancer family history using the continuous BFHS and also stratified by BFHS, dichotomized at the median.

We examined alternative modeling strategies for age at thelarche in sensitivity analyses. We considered age ≥ 13 years as late versus a referent group of 11–12 years. We categorized age at thelarche in 7 groups (≤ 9, 10, 11, 12 (referent), 13, 14 and ≥ 15 years) to explore associations with very early or very late thelarche. We also examined age at thelarche as a continuous outcome using linear regression to quantify the difference in years between exposure groups.

The proportion of missing data for early-life exposures in analyses using the entire sample ranged from < 0.1% for multiple birth to about 25% for gestational hypertensive disorders and birthweight. Gestational age at birth was missing for > 50%. Therefore, we also conducted multiple imputation analyses for all early-life exposures under the assumption that data were missing at random, conditional on the specified covariates. The imputation models included the outcome, all early-life exposures, all covariates, age at menarche, and whether the participant’s mother was alive at baseline, which was a predictor of missing early-life exposure data. We used chained equations to generate 50 imputed datasets. All participants were included in the imputation models, but analysis models were restricted to the 49,162 eligible women (Additional file 2: Fig. S2). We then ran three sets of adjusted models for early and late thelarche in the imputed datasets and combined effect estimates across datasets using Rubin’s rules [14]. Model 1 included birth cohort, race/ethnicity and childhood family income to match the complete case analysis. Model 2 additionally adjusted for maternal age and firstborn status. Model 3 mutually adjusted for early-life exposures based on a directed acyclic graph (Additional file 3: Fig. S3), with different adjustment sets for each early-life exposure.

We conducted sensitivity analyses restricted to women less than 60 years at baseline, under the hypothesis that reporting errors were likely greater for older women. In separate analyses, we restricted to women whose mothers were alive at baseline, allowing participants to potentially consult their mothers about early-life exposures, which may have improved accuracy. We excluded women who reported thelarche before age 8 years or at age 16 or later in sensitivity analyses to examine whether these extremes were driving the primary associations that we observed. Since age at menarche is correlated with age at thelarche (r = 0.6), but may be more accurately reported, we ran complementary analyses examining associations between early-life exposures and timing of menarche. We also examined associations with early thelarche (≤ 10 years) and/or early menarche (≤ 11 years) relative to experiencing neither event at an early age under the hypothesis, based on the correlation between ages at thelarche and menarche, that women who reported both events at an early age may have been more accurate in their recall of early pubertal onset than women who reported early thelarche or early menarche only. Women who reported early thelarche only have a longer time from thelarche to menarche, or pubertal tempo, and women with early menarche only have a shorter tempo, compared with women with both early thelarche and menarche. In the context of our data, relatively long or short tempo may indicate error in the recall of age at thelarche, but could also reflect true differences in tempo, which may also be biologically relevant for breast cancer risk [2].

We conducted all analyses using SAS 9.4 (SAS Institute Inc, Cary, NC).

## Results

The average age at thelarche was 12.2 years (median: 12, range: 4–20). Approximately 97% of women reported that thelarche occurred between ages 8 and 15 years, while < 0.1% reported thelarche before 8 years of age and 3% reported thelarche at age 16 or later. Early thelarche (≤ 10 years) was more common in non-Hispanic African-American/Black and Hispanic/Latina women, those born after 1960, and those who grew up in a poor household (Table 1).

**Table 1:** Distribution of age at thelarche by participant characteristics among 49,162 eligible women in the Sister Study cohort

	Age at thelarche					
	Early (≤ 10 years)		Average (11–13 years)		Late (≥ 14 years)	
	*n*	%	*n*	%	*n*	%
Number of participants	6613	13	34,030	69	8519	17
*Participant characteristics*						
Birth cohort						
1928–1939	613	10	4220	72	1045	18
1940–1949	2116	13	11,403	71	2607	16
1950–1959	2469	14	12,619	69	3186	17
1960–1974	1415	16	5788	65	1681	19
Race/ethnicity						
Non-Hispanic White	5275	13	29,141	70	7015	17
Non-Hispanic African-American/Black	754	18	2,517	61	835	20
Hispanic/Latina	422	18	1,525	65	399	17
Others^a^	162	13	847	66	270	21
Family income level growing up						
Well off	436	14	2,194	70	507	16
Middle income	3863	13	20,597	70	4899	17
Low income	1739	14	8,693	68	2331	18
Poor	575	15	2,546	65	782	20
Maternal vital status at baseline						
Alive	3198	14	15,788	69	4040	18
Deceased	3408	13	18,196	70	4465	17
Missing	7		46		14	
*Maternal pregnancy characteristics*						
Diabetes						
Any (pre-pregnancy or gestational)	77	17	307	67	74	16
None	5816	13	30,259	69	7519	17
Missing	720		3464		926	
Gestational hypertensive disorder						
Any (gestational hypertension or pre-eclampsia)	285	17	1144	67	280	16
None	4708	13	24,505	69	6086	17
Missing	1620		8381		2153	
DES use						
Yes	181	16	770	67	191	17
No	5399	13	28,273	70	6969	17
Missing	1033		4987		1359	
Smoking during pregnancy						
Yes	2201	15	10,131	68	2546	17
No	4086	13	22,236	70	5553	17
Missing	326		1663		420	
Farm exposure						
Work and residence	770	13	3977	69	983	17
Work only	88	14	409	65	130	21
Residence only	269	13	1458	71	313	15
None	5263	13	27,132	69	6774	17
Missing	223		1054		319	
Age at delivery						
< 20 years	403	17	1544	66	387	17
20–24 years	1626	14	7916	69	1970	17
25–29 years	1904	13	10,189	69	2586	18
30–34 years	1419	13	7873	70	1916	17
35–39 years	854	13	4464	70	1096	17
≥ 40 years	314	13	1672	69	444	18
Missing	93		372		120	
*Birth and infancy characteristics*						
Firstborn						
Yes	1699	15	7931	70	1664	15
No	4877	13	25,915	69	6814	18
Missing	37		184		41	
Birthweight						
< 2500 g	532	14	2505	67	692	19
2500–3999 g	4028	14	20,577	70	4900	17
≥ 4000 g	452	14	2331	70	555	17
Missing	1601		8617		2372	
Multiple birth						
Yes	182	12	1062	69	297	19
No	6430	14	32,963	69	8,221	17
Missing	1		5		1	
Gestational age at birth						
Born ≥ 1 month before due date	126	13	672	68	192	19
Born 2–4 weeks before due date	279	15	1324	70	301	16
Not born ≥ 2 weeks before due date	2784	14	13,773	69	3383	17
Missing	3424		18,261		4643	
Ever breastfed						
Yes	2993	13	15,608	70	3768	17
No	3147	14	15,923	69	4046	18
Missing	473		2499		705	
Ever fed soy formula						
Yes	179	15	797	67	219	18
No	5261	13	27,471	69	6827	17
Missing	1173		5762		1473	

Row percentages are displayed. Percentages may not add up to 100 due to rounding. Missing are not included in percentages

^a^Includes non-Hispanic Asian-Americans and Pacific Islanders (25%), non-Hispanic American Indians and Alaska natives (7%), and non-Hispanic race not specified (68%)

Early thelarche was associated with multiple pre- and postnatal exposures, while few associations were observed with late thelarche (Table 2). Maternal gestational hypertensive disorders, DES use, maternal smoking during pregnancy and having a teenage mother were each associated with a 20–30% increased odds of early thelarche in daughters. Maternal diabetes prior to pregnancy was associated with more than a 70% increased likelihood of early thelarche, but there was no association between gestational diabetes and early thelarche. Being firstborn was positively associated with early thelarche and inversely associated with late thelarche, while the opposite pattern was observed for being part of a multiple birth. Preterm birth (born ≥ 1 month before due date) was also positively associated with late thelarche. Low birthweight (< 2500 g) was positively associated with early and late thelarche relative to average birthweight (2500–3499 g), while no associations were observed for high birthweight (≥ 4000 g). A similar U-shaped pattern was observed for soy formula in infancy. Being breastfed in infancy was not associated with early thelarche, though we observed a minor decrease in the odds of late thelarche.

**Table 2:** Associations between early-life exposures and timing of thelarche in the Sister Study cohort (*N* = 49,162)

		Early thelarche (≤ 10 years)^a,b^		Late thelarche (≥ 14 years)^a,b^	
	*N*	OR	95% CI	OR	95% CI
*Maternal pregnancy characteristics*					
Diabetes					
Any	458	1.20	0.93, 1.54	0.91	0.70, 1.17
Gestational diabetes	215	0.79	0.52, 1.21	0.87	0.60, 1.25
Pre-pregnancy diabetes	223	1.72	1.24, 2.38	0.88	0.60, 1.29
None	43,594	1	Ref	1	Ref
Gestational hypertensive disorder					
Any	1709	1.25	1.09, 1.43	0.96	0.84, 1.10
Pre-eclampsia	887	1.32	1.10, 1.58	0.99	0.83, 1.19
Gestational hypertension	626	1.12	0.90, 1.40	0.80	0.64, 1.01
None	35,299	1	Ref	1	Ref
DES use					
Yes	1142	1.23	1.04, 1.45	1.02	0.86, 1.19
No	40,641	1	Ref	1	Ref
Smoking during pregnancy					
Yes	14,878	1.20	1.13, 1.27	1.02	0.97, 1.08
No	31,875	1	Ref	1	Ref
Farm exposure					
Work and residence	5730	1.00	0.92, 1.09	0.94	0.87, 1.02
Work only	627	1.06	0.84, 1.33	1.21	0.99, 1.48
Residence only	2040	0.98	0.86, 1.12	0.86	0.76, 0.98
None	39,169	1	Ref	1	Ref
Age at delivery					
< 20 years	2334	1.30	1.16, 1.47	0.93	0.82, 1.05
20–24 years	11,512	1.09	1.01, 1.17	0.97	0.91, 1.03
25–29 years	14,679	1	Ref	1	Ref
30–34 years	11,208	0.95	0.88, 1.02	0.95	0.89, 1.01
35–39 years	6414	0.99	0.90, 1.08	0.94	0.87, 1.02
≥ 40 years	2430	0.95	0.83, 1.08	1.00	0.89, 1.12
*Birth and infancy characteristics*					
Firstborn					
Yes	11,294	1.25	1.17, 1.33	0.84	0.79, 0.89
No	37,606	1	Ref	1	Ref
Birthweight					
< 2500 g	3729	1.06	0.96, 1.17	1.15	1.05, 1.25
2500–3999 g	29,505	1	Ref	1	Ref
≥ 4000 g	3,338	1.00	0.90, 1.11	0.99	0.90, 1.09
Multiple birth					
Yes	1541	0.87	0.74, 1.02	1.11	0.98, 1.27
No	47,614	1	Ref	1	Ref
Gestational age at birth					
Born ≥ 1 month before due date	990	0.91	0.75, 1.11	1.16	0.99, 1.37
Born 2–4 weeks before due date	1904	1.06	0.92, 1.21	0.93	0.81, 1.06
Not born ≥ 2 weeks before due date	19,940	1	Ref	1	Ref
Ever breastfed					
Yes	22,369	0.98	0.93, 1.04	0.95	0.90, 1.00
No	23,116	1	Ref	1	Ref
Ever fed soy formula					
Yes	1195	1.10	0.93, 1.30	1.07	0.92, 1.25
No	39,559	1	Ref	1	Ref

^a^Adjusted for birth cohort, race/ethnicity and childhood family income

^b^Referent group is thelarche at 11–13 years

Associations were similar in models adjusted for relative weight at 10 years of age, except for birthweight (Additional file 4: Table S1). Adjusting for relative weight, low birthweight was associated with early (OR = 1.16, 95% CI 1.05–1.28), but not late (OR = 1.03, 95% CI 0.94–1.13) thelarche. In addition, high birthweight was inversely associated with early (OR = 0.90, 95% CI 0.81–1.00) and positively associated with late (OR = 1.09, 95% CI 0.99–1.21) thelarche. Patterns were similar within strata of childhood weight (Additional file 5: Table S2, birthweight p for heterogeneity by childhood weight = 0.99).

Patterns of association were similar across strata of birth cohort (Additional file 6: Table S3), race/ethnicity (Additional file 7: Table S4), childhood family income (Additional file 8: Table S5) and extent of breast cancer family history (Additional file 9: Table S6). The positive association of soy formula in infancy with early thelarche was only observed among women born in 1960–1974 (OR = 1.34, 95% CI 1.03–1.74), non-Hispanic African-American/Black women (OR = 1.63, 95% CI 1.02–2.60) and women who grew up in a poor family (OR = 1.70, 95% CI 1.00–2.90), though the interaction was statistically significant for childhood family income only (*p* = 0.02).

Results were similar when thelarche at 11–12 years was used as the referent group (data not shown). Associations were generally stronger in magnitude when we considered very early thelarche (≤ 9 years) (Additional file 10: Table S7). U-shaped associations of low birthweight and soy formula with age at thelarche were more prominent in the model that included 7 thelarche categories. The inference for most early-life exposures and timing of thelarche was similar when age at thelarche was modeled continuously using linear regression (Additional file 10: Table S7). Exceptions include DES use, which was not associated with thelarche when modeled continuously, along with low birthweight and soy formula, which were positively associated with both early and late thelarche in the polytomous models.

Results were nearly identical in multiple imputation analyses (Additional file 11: Table S8). The inference also was unchanged in analyses limited to either women younger than 60 years or women whose mother was still living at baseline (data not shown), except that pre-pregnancy diabetes was no longer associated with early thelarche after excluding women whose mother was deceased at baseline. Results were similar for all exposures in sensitivity analyses excluding women with extremely early (< 8 years) or late (≥ 16 years) thelarche (data not shown). Patterns were similar when we examined early-life exposures in relation to age at menarche as a marker of pubertal timing instead of thelarche (Additional file 12: Table S9). Associations of early-life exposures with early thelarche and early menarche were generally stronger in magnitude than associations with early thelarche or early menarche only, except for birthweight and gestational age (Additional file 13: Table S10).

## Discussion

Multiple pre- and postnatal exposures were associated with early thelarche in a diverse, nationwide cohort of women with a family history of breast cancer. Associations did not meaningfully vary by birth cohort, race/ethnicity, socioeconomic status, or extent of breast cancer family history. Our findings support the hypothesis that the early-life environment influences the timing of pubertal breast development, especially in sub-groups who experience differential burdens of early thelarche.

Maternal pre-pregnancy obesity is a risk factor for developing gestational hypertensive disorders in pregnancy [15] and has also been associated with earlier thelarche in daughters in contemporary cohorts [16, 17]. We did not collect data on maternal pre-pregnancy body mass index (BMI), which may underlie the observed associations of gestational hypertensive disorders and maternal pre-pregnancy diabetes with earlier thelarche. In the Danish National Birth Cohort (DNBC), crude differences in mean age at thelarche in daughters of women with gestational hypertensive disorders compared to daughters of women with normotensive pregnancies were attenuated after adjustment for maternal pre-pregnancy BMI and other factors [18]. In contrast, a Norwegian case–control study found that daughters exposed to pre-eclamptic pregnancies were less likely to experience thelarche by 10.8 years of age than daughters of normotensive mothers, independent of maternal BMI, but only among exclusively breastfed girls [19].

We did not observe an association between gestational diabetes and age at thelarche, consistent with two prospective studies of girls enrolled in the Kaiser Permanente Northern California (KPNC) health system [17, 20]. In DNBC, there was no difference in mean age at thelarche in daughters of women with gestational diabetes, type 1 diabetes or type 2 diabetes after adjustment for maternal confounders, including pre-pregnancy BMI [21]. A prior, small study (n = 310 girls) within the DNBC found that daughters of women with gestational diabetes experienced earlier thelarche than controls, but did not adjust for maternal confounders [22].

We found that women exposed in utero to DES, a potent synthetic estrogen, were more likely to experience early thelarche. Earlier vaginal opening, an estrogen-mediated marker of pubertal onset analogous to thelarche in humans [23, 24], has been observed in rodents exposed to DES during gestation [25]. A study of 30 DES-exposed daughters and 30 controls found no difference in mean age at thelarche associated with DES [26]. While strengths of that study were the use of medical record-confirmed history of prenatal DES exposure and shorter recall time for age at thelarche (recalled at ages 17–30), the small sample size, women with abnormal Pap smears as controls, and lack of control for confounding could explain the lack of association with age at thelarche. We did not observe an association when age at thelarche was modeled as a continuous outcome. This is in line with previous studies, including in our cohort and a cohort with record-confirmed DES exposure, that observed an increased risk of very early menarche (≤ 10 years) in DES daughters [27, 28], while others did not observe a difference in mean age at menarche [26, 29, 30].

Women who were fed soy formula in infancy, which includes high concentrations of phytoestrogens, including genistein [31, 32], were more likely to experience very early and very late thelarche. The effects of phytoestrogens on reproductive development in animal and human studies have varied by timing and dose of exposure [33]. Experimental evidence in mice has observed opposing effects on pubertal onset associated with neonatal genistein administration: mice administered low-dose genistein had earlier vaginal opening, while those administered a high dose had later opening [34]. Three small prospective studies of girls fed soy formula in infancy did not observe differences in breast bud volume [35] or timing of pubertal breast development [36, 37] compared with those fed cow’s milk formula or breastmilk. The association we observed with early thelarche was specific to African-American/Black women and those who grew up in poor households, two groups at increased risk of early thelarche [4, 9]. Our results may be explained by residual confounding, as families who choose soy formula may differ in other ways that affect timing of thelarche. We did not observe later thelarche in women who were breastfed in infancy, as has been observed in some [38–40], but not all [41], prospective cohorts.

Higher in utero estrogen exposure also may explain the earlier age at thelarche we observed in firstborn daughters, as maternal estrogens are higher in first compared to subsequent full-term pregnancies [42]. Higher cord blood estrogen concentrations have also been observed in firstborn compared with later born children [43]. Being firstborn was associated with earlier thelarche in the Avon Longitudinal Study of Parents and Children (ALSPAC) cohort [44] and earlier pubarche, but not thelarche, in the Hong Kong Children of 1997 birth cohort [45]. We also observed earlier thelarche in daughters of teenage mothers, who are more likely to be firstborn, though these associations were independent of one another.

While cigarette smoke is thought to be anti-estrogenic, it includes thousands of chemicals, including reproductive and developmental toxicants and endocrine disruptors, that may affect pubertal timing [46]. Although rates of smoking during pregnancy have decreased in the USA in more recent birth cohorts [47], smoking during pregnancy is still prevalent in many countries [48]. About one-third of the women in our cohort reported that their mother smoked during pregnancy, which was associated with earlier thelarche. Two studies conducted in prospective European cohorts, in which approximately 20–30% of girls were exposed to maternal smoking during pregnancy, also observed associations with earlier thelarche [44, 49]. In a US cohort of girls born in the late 1990s in which < 10% were exposed to prenatal smoke, no association was observed overall with age at thelarche [50].

Low birthweight was associated with early and late thelarche, while being born more than a month early and being part of a multiple gestation, conditions associated with low birthweight, were both associated with later thelarche. Findings from previous studies examining birthweight, size for gestational age and/or preterm birth with age at thelarche have been inconsistent [44, 51–60]. High birthweight is associated with childhood obesity [61], a risk factor for early thelarche [4]. In our data, women with high birthweight were more likely to report that they were heavier than their peers at age 10, while women with low birthweight were more likely to report that they were lighter than their peers; relative weight at age 10 was inversely associated with age at thelarche (data not shown). However, low birthweight infants are more likely to experience rapid postnatal growth [62], which is also associated with earlier thelarche [44, 60]. We hypothesize that the U-shaped association that we observed overall with low birthweight may reflect modification by postnatal growth patterns. Postnatal growth may also explain the change in the association between birthweight and age at thelarche when we controlled for childhood body size by adjustment or stratification. High birthweight babies may regress toward the mean after birth through slower or catch-down growth [63]. While we do not have data to examine the influence of postnatal growth directly, our finding that low birthweight was positively associated with early thelarche while high birthweight was associated with late thelarche in models stratified by childhood body size suggest that, among girls of similar body size at age 10, those that grew more rapidly between birth and age 10 experienced earlier thelarche, while those that grew more slowly between birth and age 10 experienced later thelarche. Alternatively, other factors that influence fetal growth could underlie the associations we observed between birthweight and age at thelarche.

The average age at thelarche of 12 years in our cohort was about a year later than what has been reported in prospective studies of women born around the same time [64]. The distribution of age at thelarche in our cohort was slightly right-skewed, which also suggests that women in our cohort may have recalled a later age at thelarche than when it truly occurred. While some misreporting of recalled age at thelarche is likely, categorizing age at thelarche as early, average and late may have minimized measurement error, as has been observed for age at menarche [65]. The prevalence of early thelarche increased in successive birth cohorts and early thelarche was more common in Black and Hispanic women. These demographic differences are consistent with prospective thelarche data [4, 6], suggesting that our recalled measure likely captured women who experienced thelarche relatively early compared to their peers, even if there was error in the recall of the absolute age. Associations of early-life exposures with early and late age at menarche, which previous studies have shown is reliably reported into adulthood [66] and is correlated with age at thelarche [64], were similar to the associations we observed with early and late thelarche, which suggests that measurement error in recalled age at thelarche is unlikely to explain our findings. In addition, associations were generally stronger in magnitude for women who reported both early thelarche and early menarche, suggesting that misreporting of age at thelarche may have biased the results of our primary analyses of early thelarche toward the null. An alternate interpretation of this analysis is that stronger associations of early-life exposures with early ages at thelarche and menarche, compared with associations of early-life exposures with early thelarche without early menarche, reflect an association of these exposures with shorter pubertal tempo. However, potential measurement error in addition to imprecision in the assessment of ages at both thelarche and menarche, recalled to the nearest year, makes it difficult to accurately assess pubertal tempo using retrospective data, so it is not clear the extent to which this analysis captures true differences in tempo.

Strengths of this study include the large sample size and wide range of pre- and postnatal exposures. We were able to examine past exposures that are no longer used at all or as frequently, such as DES use and smoking during pregnancy, but are informative for current exposures to endocrine-disrupting chemicals [67]. We were limited by recalled data on early-life exposures, which may be reported with error. Participants were provided with a prepaid phone card and encouraged to contact their mothers or other relatives for assistance in completing the early-life exposure information, but we do not know how many women did. In a validation study, a sample of 1,800 mothers of participants under 60 years of age at enrollment completed a similar questionnaire on pregnancy-related factors. Agreement between daughter’s and mother’s report was good for most exposures, with kappas ranging from 0.6 (pre-eclampsia) to 0.9 (birth order, maternal age).

Our results may be subject to confounding by other unmeasured factors such as pre-pregnancy BMI, gestational weight gain, and genetic influences, as well as residual confounding or variation by socioeconomic factors. While we consider the diversity of our cohort to be a strength, we had reduced precision in stratified analyses in some groups, such as racial/ethnic minorities. Women in our cohort have at least one sister with breast cancer, and have, on average, approximately twice the risk of breast cancer as women without a first-degree family history [68]. While we observed no differences by extent of familial risk, our results may not be generalizable to women without a family history of breast cancer. Nonetheless, it is important to examine risk factors for early thelarche, a breast cancer risk factor, among women at increased risk of breast cancer due to their family history as these women may derive the most benefit from early-life interventions to reduce their lifetime risk of breast cancer.

## Conclusions

Our findings suggest that the early-life environment influences breast development and may influence the risk of breast cancer by altering the timing of pubertal onset. Our results also support the hypothesis that environmental factors acting early in life, including maternal pregnancy complications and exposure to endocrine-disrupting chemicals, contribute to the secular decline in age at thelarche, which may lead to future increases in breast cancer incidence.

## Supplementary Information

**Additional file 1: Fig. S1**. Distribution of age at thelarche in the analytic sample

**Additional file 2: Fig. S2**. Flowchart of Sister Study participants included in analytic sample

**Additional file 3: Fig. S3**. Directed acyclic graph of hypothesized associations between early-life exposures and age at thelarche

**Additional file 4: Table S1**. Associations between early-life exposures and timing of thelarche in the Sister Study cohort additionally adjusted for relative weight at age 10 (*N* = 49,033)

**Additional file 5: Table S2**. Associations between early-life exposures and timing of thelarche in the Sister Study cohort by relative weight at age 10 (*N* = 49,033)

**Additional file 6: Table S3**. Associations between early-life exposures and timing of thelarche in the Sister Study cohort by birth cohort (*N* = 49,162)

**Additional file 7: Table S4**. Associations between early-life exposures and timing of thelarche in the Sister Study cohort by race/ethnicity (*N* = 47,883)

**Additional file 8: Table S5**. Associations between early-life exposures and timing of thelarche in the Sister Study cohort by qualitative childhood family income (*N* = 49,162)

**Additional file 9: Table S6**. Associations between early-life exposures and timing of thelarche in the Sister Study cohort by Bayesian family history score (BFHS) (*N* = 49,151)

**Additional file 10: Table S7**. Associations between early-life exposures and age at thelarche using alternate characterizations of age at thelarche in the Sister Study cohort (*N* = 49,162)

**Additional file 11: Table S8**. Associations between early-life exposures and timing of thelarche in the Sister Study cohort with multiple imputation of missing early-life exposure data (*N* = 49,162)

**Additional file 12: Table S9**. Associations between early-life exposures and timing of menarche in the Sister Study cohort (*N* = 49,130)

**Additional file 13: Table S10**. Associations between early-life exposures and early thelarche (≤ 10 years) and/or early menarche (≤ 11 years) in the Sister Study cohort (*N* = 49,130)

## Data Availability

Requests for data, including the data used in this manuscript, are welcome. De-identified data are made available upon request as described on the study website (https://sisterstudy.niehs.nih.gov/English/data-requests.htm). The data sharing policy was developed to protect the privacy of study participants and is consistent with study informed consent documents as approved by the NIEHS Institutional Review Board.

## Abbreviations

BFHS: Bayesian family history score
BMI: Body mass index
DES: Diethylstilbestrol
CI: Confidence interval
OR: Odds ratio
