# Supplementary figures and images for: Early-life exposures and age at thelarche in the Sister Study cohort
Source: Breast Cancer Res. 2021 Dec 11;23:111. doi: 10.1186/s13058-021-01490-z (PMC8666031; doi:10.1186/s13058-021-01490-z)

**Figure S1.** Distribution of age at thelarche in the analytic sample

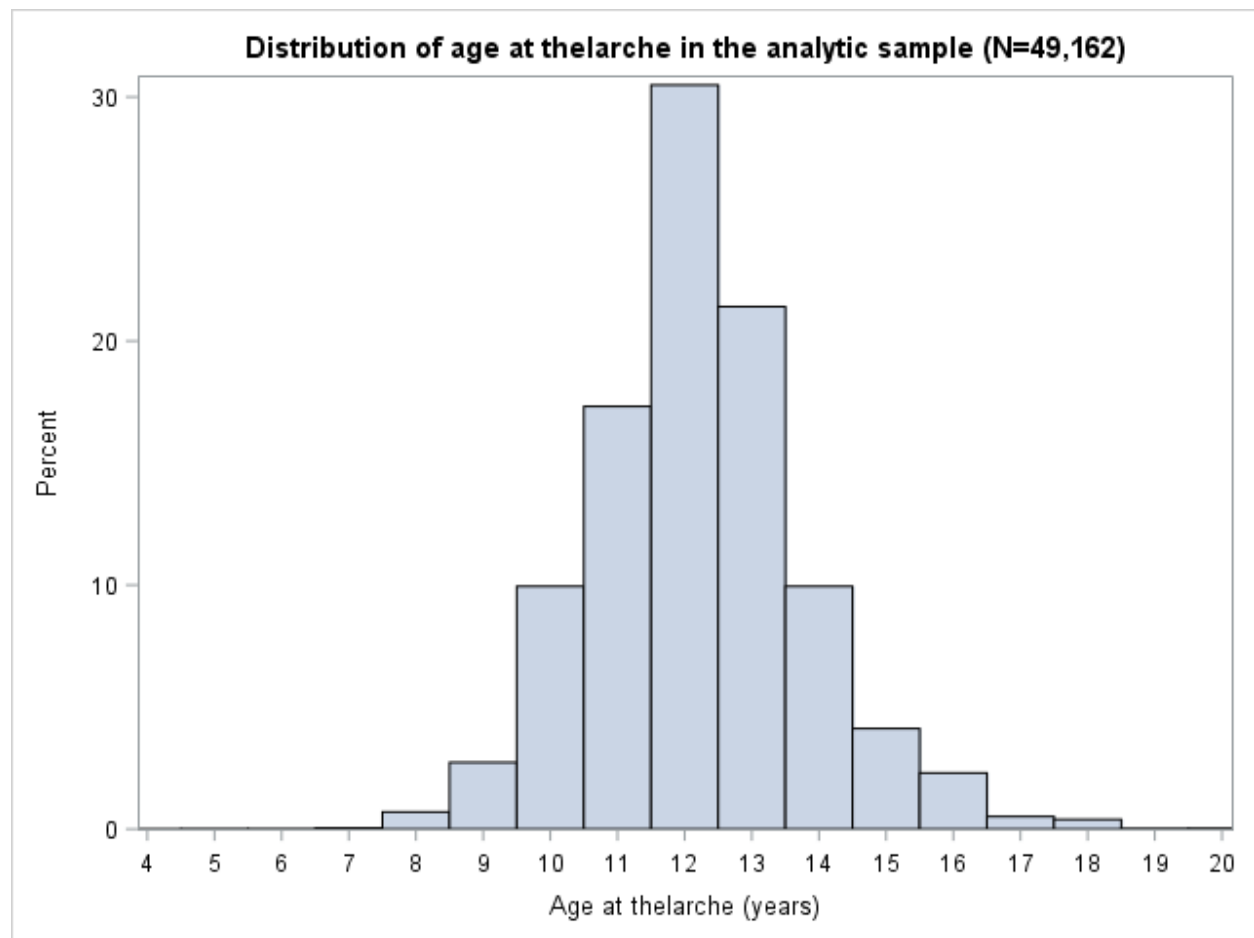

Supplement: Supplementary file 1 — Additional file 1: Fig. S1. Distribution of age at thelarche in the analytic sample [file 13058_2021_1490_MOESM1_ESM.pdf]

**Figure S2.** Flow chart of Sister Study participants included in analytic sample

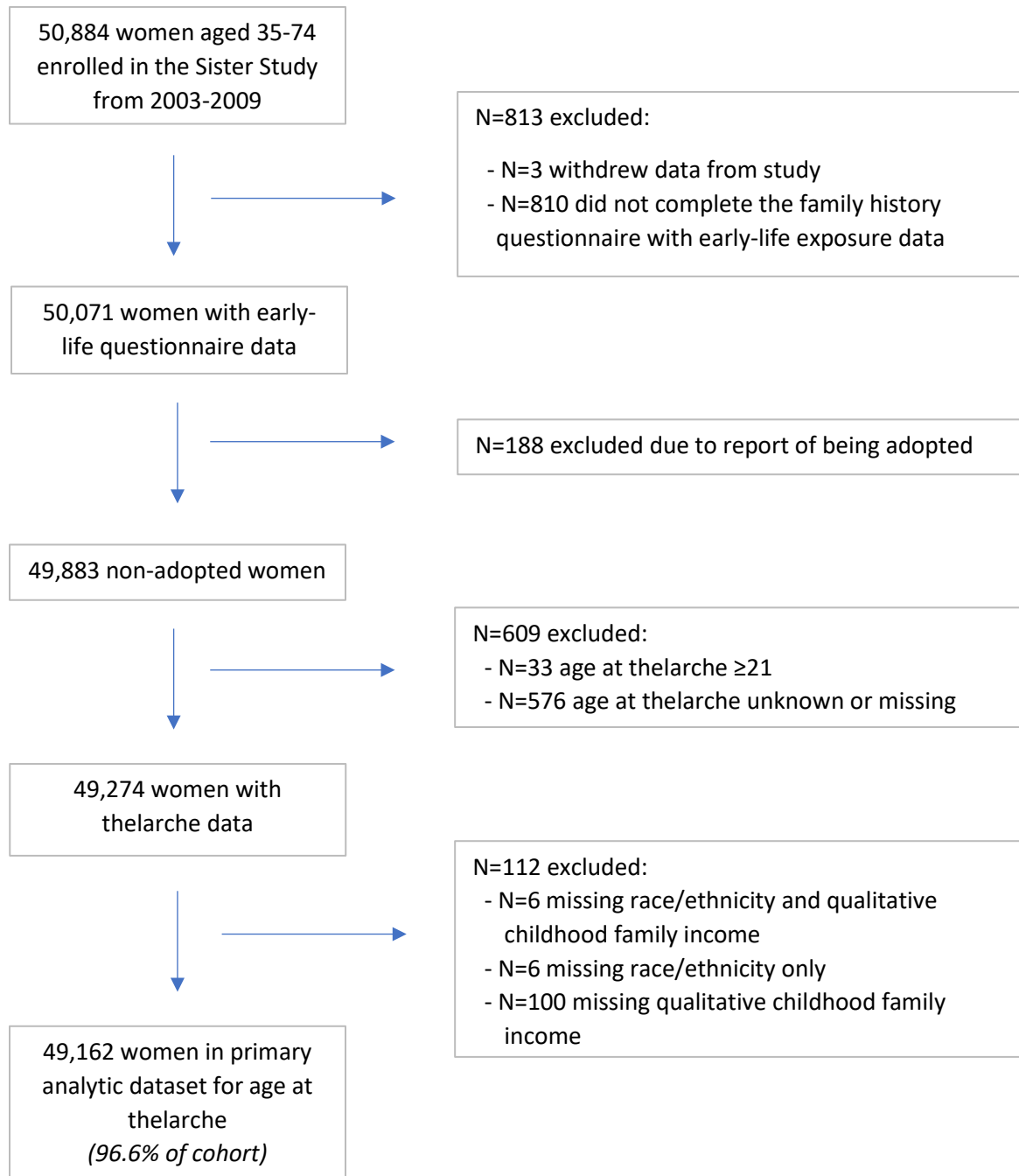

Supplement: Supplementary file 2 — Additional file 2: Fig. S2. Flowchart of Sister Study participants included in analytic sample [file 13058_2021_1490_MOESM2_ESM.pdf]
