# Supplementary material for: Early-life exposures and age at thelarche in the Sister Study cohort
Source: Breast Cancer Res. 2021 Dec 11;23:111. doi: 10.1186/s13058-021-01490-z (PMC8666031; doi:10.1186/s13058-021-01490-z)

Figure S3. Directed acyclic graph of hypothesized associations between early-life exposures and age at thelarche

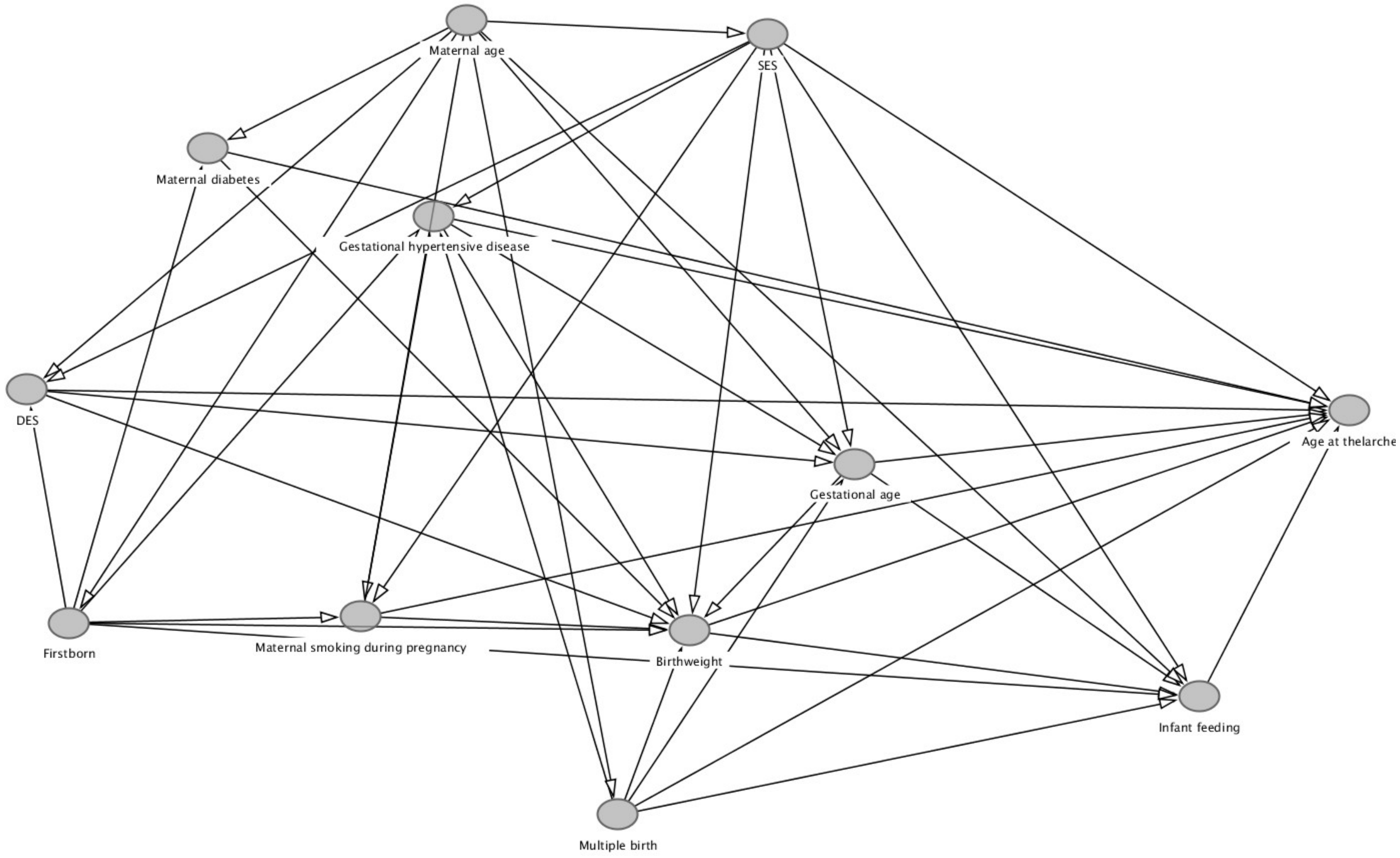

Supplement: Supplementary file 3 — Additional file 3: Fig. S3. Directed acyclic graph of hypothesized associations between early-life exposures and age at thelarche [file 13058_2021_1490_MOESM3_ESM.pdf]
