# Supplementary material for: Early-life exposures and age at thelarche in the Sister Study cohort
Source: Breast Cancer Res. 2021 Dec 11;23:111. doi: 10.1186/s13058-021-01490-z (PMC8666031; doi:10.1186/s13058-021-01490-z)
Supplement: Supplementary file 4 — Additional file 4: Table S1. Associations between early-life exposures and timing of thelarche in the Sister Study cohort additionally adjusted for relative weight at age 10 (N = 49,033) [file 13058_2021_1490_MOESM4_ESM.pdf]

Table S1. Associations between early-life exposures and timing of thelarche in the Sister Study cohort additionally adjusted for relative weight at age 10 (N=49,033)[a]

	Early thelarche (≤10 years)[b,c]		Late thelarche (≥14 years)[b,c]	
	OR	95% CI	OR	95% CI
Maternal pregnancy characteristics				
Diabetes				
Any	1.18	0.91, 1.52	0.90	0.70, 1.17
Gestational diabetes	*0.81*	*0.53, 1.24*	*0.86*	*0.59, 1.25*
Pre-pregnancy diabetes	*1.65*	*1.18, 2.30*	*0.87*	*0.59, 1.28*
None	1	Ref	1	Ref
Gestational hypertensive disorder				
Any	1.23	1.07, 1.41	0.95	0.83, 1.09
Pre-eclampsia	*1.33*	*1.11, 1.61*	*0.96*	*0.80, 1.16*
Gestational hypertension	*1.08*	*0.86, 1.35*	*0.81*	*0.64, 1.02*
None	1	Ref	1	Ref
DES use				
Yes	1.24	1.05, 1.47	0.99	0.84, 1.17
No	1	Ref	1	Ref
Smoking during pregnancy				
Yes	1.16	1.10, 1.23	1.04	0.98, 1.10
No	1	Ref	1	Ref
Farm exposure				
Work and residence	1.00	0.92, 1.09	0.96	0.89, 1.04
Work only	1.04	0.82, 1.32	1.21	0.99, 1.49
Residence only	0.99	0.87, 1.14	0.85	0.75, 0.96
None	1	Ref	1	Ref
Age at delivery				
<20 years	1.37	1.21, 1.55	0.89	0.79, 1.01
20-24 years	1.11	1.03, 1.20	0.96	0.90, 1.03
25-29 years	1	Ref	1	Ref
30-34 years	0.93	0.86, 1.00	0.95	0.89, 1.02
35-39 years	0.96	0.88, 1.05	0.95	0.88, 1.03
≥40 years	0.92	0.81, 1.05	1.00	0.89, 1.13
Birth and infancy characteristics				
Firstborn				
Yes	1.27	1.19, 1.35	0.84	0.79, 0.89
No	1	Ref	1	Ref
Birthweight				
<2500g	1.16	1.05, 1.28	1.03	0.94, 1.13
2500g-3999g	1	Ref	1	Ref

≥4000g	0.90	0.81, 1.00	1.09	0.99, 1.21
Multiple birth				
Yes	0.92	0.79, 1.09	1.08	0.94, 1.23
No	1	Ref	1	Ref
Gestational age at birth				
Born ≥1 month before due date	0.92	0.75, 1.12	1.09	0.92, 1.29
Born 2-4 weeks before due date	1.06	0.92, 1.21	0.92	0.81, 1.05
Not born ≥2 weeks before due date	1	Ref	1	Ref
Ever breastfed				
Yes	1.00	0.94, 1.06	0.95	0.90, 1.00
No	1	Ref	1	Ref
Ever fed soy formula				
Yes	1.10	0.93, 1.30	1.06	0.91, 1.24
No	1	Ref	1	Ref

[a]129 women with missing data on weight at age 10 were excluded from this analysis.

[b]Adjusted for birth cohort, race/ethnicity, childhood family income and relative weight at age 10.

[c]Referent group is thelarche at 11-13 years
