# Supplementary material for: Early-life exposures and age at thelarche in the Sister Study cohort
Source: Breast Cancer Res. 2021 Dec 11;23:111. doi: 10.1186/s13058-021-01490-z (PMC8666031; doi:10.1186/s13058-021-01490-z)
Supplement: Supplementary file 5 — Additional file 5: Table S2. Associations between early-life exposures and timing of thelarche in the Sister Study cohort by relative weight at age 10 (N = 49,033) [file 13058_2021_1490_MOESM5_ESM.pdf]

Table S2. Associations between early-life exposures and timing of thelarche in the Sister Study cohort by relative weight at age 10 (N=49,033)[a]

	Lighter (n=17,217)				Same (n=22,910)				Heavier (n=8906)				
	Early (≤10 years)[b,c]		Late (≥14 years)[b,c]		Early (≤10 years)[b,c]		Late (≥14 years)[b,c]		Early (≤10 years)[b,c]		Late (≥14 years)[b,c]		
	OR	95% CI	OR	95% CI	OR	95% CI	OR	95% CI	OR	95% CI	OR	95% CI	P-het[d]
Maternal pregnancy characteristics													
Diabetes													0.45
Any	1.34	0.80, 1.22	0.88	0.60, 1.27	0.93	0.61, 1.42	1.02	0.69, 1.51	1.39	0.89, 2.17	0.60	0.21, 1.65	
None	1	Ref	1	Ref	1	Ref	1	Ref	1	Ref	1	Ref	
Gestational hypertensive disorder													0.32
Any	1.32	1.00, 1.74	0.98	0.81, 1.18	1.19	0.96, 1.46	0.83	0.65, 1.04	1.24	0.97, 1.57	1.35	0.91, 1.98	
None	1	Ref	1	Ref	1	Ref	1	Ref	1	Ref	1	Ref	
DES use													0.69
Yes	1.18	0.82, 1.69	0.89	0.71, 1.13	1.25	0.97, 1.60	1.05	0.81, 1.36	1.32	0.98, 1.78	1.32	0.81, 2.16	
No	1	Ref	1	Ref	1	Ref	1	Ref	1	Ref	1	Ref	
Smoking during pregnancy													0.78
Yes	1.20	1.06, 1.36	1.05	0.97, 1.13	1.17	1.08, 1.28	1.05	0.97, 1.15	1.11	1.00, 1.23	0.95	0.80, 1.14	
No	1	Ref	1	Ref	1	Ref	1	Ref	1	Ref	1	Ref	
Farm exposure													0.50
Work and residence	1.00	0.83, 1.21	0.98	0.88, 1.10	0.94	0.83, 1.07	0.90	0.80, 1.02	1.11	0.95, 1.31	1.18	0.91, 1.53	
Work only	0.85	0.51, 1.44	1.16	0.87, 1.55	1.21	0.86, 1.71	1.28	0.93, 1.77	0.96	0.63, 1.46	1.21	0.64, 2.29	
Residence only	1.03	0.78, 1.36	0.79	0.66, 0.95	0.87	0.71, 1.08	0.89	0.73, 1.09	1.17	0.92, 1.49	1.01	0.66, 1.54	
None	1	Ref	1	Ref	1	Ref	1	Ref	1	Ref	1	Ref	
Age at delivery													0.70
<20 years	1.54	1.22, 1.95	0.94	0.79, 1.11	1.37	1.14, 1.64	0.87	0.72, 1.06	1.19	0.93, 1.52	0.77	0.48, 1.23	
20-24 years	1.02	0.87, 1.20	0.95	0.87, 1.05	1.14	1.03, 1.27	0.98	0.88, 1.08	1.14	1.00, 1.30	0.90	0.72, 1.14	
25-29 years	1	Ref	1	Ref	1	Ref	1	Ref	1	Ref	1	Ref	
30-34 years	0.90	0.76, 1.07	0.97	0.88, 1.07	0.96	0.86, 1.08	0.92	0.82, 1.02	0.90	0.79, 1.03	1.02	0.82, 1.26	
35-39 years	1.04	0.86, 1.27	0.95	0.85, 1.07	0.95	0.83, 1.09	0.98	0.87, 1.11	0.92	0.79, 1.03	0.88	0.68, 1.15	
≥40 years	1.14	0.87, 1.49	1.04	0.88, 1.22	0.92	0.75, 1.12	0.95	0.79, 1.14	0.81	0.64, 1.02	1.13	0.80, 1.59	
Birth and infancy characteristics													
Firstborn													0.65
Yes	1.25	1.09, 1.43	0.88	0.80, 0.95	1.27	1.16, 1.40	0.80	0.73, 0.89	1.27	1.13, 1.42	0.77	0.62, 0.96	

No	1	Ref	1	Ref	1	Ref	1	Ref	1	Ref	1	Ref	
Birthweight													0.99
<2500g	1.18	0.98, 1.41	1.02	0.91, 1.15	1.17	1.01, 1.37	1.06	0.90, 1.24	1.11	0.91, 1.35	1.04	0.73, 1.48	
2500g-3999g	1	Ref	1	Ref	1	Ref	1	Ref	1	Ref	1	Ref	
≥4000g	0.81	0.60, 1.08	1.10	0.94, 1.29	0.96	0.82, 1.13	1.09	0.94, 1.26	0.87	0.73, 1.04	1.09	0.83, 1.44	
Multiple birth													0.92
Yes	1.00	0.72, 1.37	1.07	0.89, 1.29	0.85	0.67, 1.09	1.07	0.87, 1.32	1.00	0.73, 1.36	1.17	0.72, 1.90	
No	1	Ref	1	Ref	1	Ref	1	Ref	1	Ref	1	Ref	
Gestational age at birth													0.16
Born ≥1 month before due date	0.75	0.50, 1.14	0.98	0.78, 1.23	1.02	0.74, 1.40	1.37	1.04, 1.81	0.95	0.68, 1.32	0.89	0.49, 1.60	
Born 2-4 weeks before due date	1.30	0.98, 1.72	1.01	0.84, 1.22	0.96	0.77, 1.18	0.78	0.62, 0.97	1.06	0.83, 1.35	1.13	0.76, 1.69	
Not born ≥2 weeks before due date	1	Ref	1	Ref	1	Ref	1	Ref	1	Ref	1	Ref	
Ever breastfed													0.06
Yes	1.03	0.91, 1.17	0.9	0.83, 0.97	1.04	0.95, 1.13	0.98	0.91, 1.07	0.93	0.84, 1.03	1.09	0.91, 1.29	
No	1	Ref	1	Ref	1	Ref	1	Ref	1	Ref	1	Ref	
Ever fed soy formula													0.76
Yes	1.10	0.76, 1.57	1.09	0.87, 1.35	1.17	0.92, 1.50	0.99	0.77, 1.28	0.99	0.73, 1.33	1.24	0.78, 1.96	
No	1	Ref	1	Ref	1	Ref	1	Ref	1	Ref	1	Ref	

[a]129 women with missing data on weight at age 10 were excluded from this analysis.

[b]Adjusted for birth cohort, race/ethnicity, and childhood family income

[c]Referent group is thelarche at 11-13 years

[d]P for heterogeneity calculated from a likelihood ratio test of nested models
