# Supplementary material for: Early-life exposures and age at thelarche in the Sister Study cohort
Source: Breast Cancer Res. 2021 Dec 11;23:111. doi: 10.1186/s13058-021-01490-z (PMC8666031; doi:10.1186/s13058-021-01490-z)
Supplement: Supplementary file 6 — Additional file 6: Table S3. Associations between early-life exposures and timing of thelarche in the Sister Study cohort by birth cohort (N = 49,162) [file 13058_2021_1490_MOESM6_ESM.pdf]

Table S3. Associations between early-life exposures and timing of thelarche in the Sister Study cohort by birth cohort (N=49,162)

	1928-1939 (n=5878)				1940-1949 (n=16,126)				1950-1959 (n=18,274)				1960-1974 (n=8884)				
	Early (≤10 years)[a,b]		Late (≥14 years)[a,b]		Early (≤10 years)[a,b]		Late (≥14 years)[a,b]		Early (≤10 years)[a,b]		Late (≥14 years)[a,b]		Early (≤10 years)[a,b]		Late (≥14 years)[a,b]		
	OR	95% CI	OR	95% CI	OR	95% CI	OR	95% CI	OR	95% CI	OR	95% CI	OR	95% CI	OR	95% CI	P-het[c]
Maternal pregnancy characteristics																	
Diabetes																	0.59
Any	1.37	0.56, 3.36	0.70	0.27, 1.83	1.71	1.07, 2.72	1.10	0.67, 1.81	1.14	0.76, 1.73	0.86	0.56, 1.30	0.86	0.52, 1.43	0.89	0.55, 1.42	
None	1	Ref	1	Ref	1	Ref	1	Ref	1	Ref	1	Ref	1	Ref	1	Ref	
Gestational hypertensive disorder																	0.81
Any	1.29	0.80, 2.08	1.10	0.73, 1.64	1.21	0.94, 1.57	0.96	0.74, 1.24	1.30	1.06, 1.61	0.86	0.69, 1.07	1.21	0.92, 1.59	1.10	0.84, 1.44	
None	1	Ref	1	Ref	1	Ref	1	Ref	1	Ref	1	Ref	1	Ref	1	Ref	
DES use[d]																	0.59
Yes	-	-	-	-	1.41	1.02, 1.95	1.06	0.76, 1.48	1.12	0.89, 1.41	1.06	0.86, 1.31	1.35	0.94, 1.93	0.87	0.59, 1.27	
No	-	-	-	-	1	Ref	1	Ref	1	Ref	1	Ref	1	Ref	1	Ref	
Smoking during pregnancy																	0.85
Yes	1.40	1.14, 1.72	1.03	0.86, 1.24	1.17	1.06, 1.30	1.02	0.93, 1.13	1.19	1.08, 1.30	1.02	0.94, 1.11	1.18	1.04, 1.34	1.04	0.93, 1.18	
No	1	Ref	1	Ref	1	Ref	1	Ref	1	Ref	1	Ref	1	Ref	1	Ref	
Farm exposure																	0.14
Work and residence	1.14	0.91, 1.43	1.01	0.85, 1.21	1.02	0.89, 1.17	1.01	0.89, 1.15	0.85	0.73, 0.99	0.83	0.72, 0.95	1.19	0.95, 1.49	0.91	0.72, 1.14	
Work only	0.90	0.38, 2.11	1.10	0.60, 2.02	1.00	0.66, 1.51	1.27	0.90, 1.79	1.33	0.93, 1.88	1.31	0.96, 1.81	0.75	0.42, 1.33	0.96	0.58, 1.58	
Residence only	1.39	0.98, 1.96	0.86	0.62, 1.18	0.85	0.68, 1.06	0.79	0.64, 0.97	0.93	0.73, 1.17	0.87	0.71, 1.08	1.18	0.84, 1.67	1.08	0.78, 4.51	
None	1	Ref	1	Ref	1	Ref	1	Ref	1	Ref	1	Ref	1	Ref	1	Ref	
Age at delivery																	0.23
<20 years	1.95	1.39, 2.75	1.29	0.94, 1.76	1.32	1.07, 1.62	0.83	0.66, 1.03	1.24	1.01, 1.51	0.90	0.74, 1.09	1.10	0.81, 1.48	0.89	0.66, 1.20	
20-24 years	1.12	0.90, 1.40	0.93	0.78, 1.12	1.04	0.92, 1.18	1.02	0.91, 1.14	1.14	1.01, 1.28	0.93	0.83, 1.04	1.07	0.90, 1.27	1.00	0.85, 1.17	
25-29 years	1	Ref	1	Ref	1	Ref	1	Ref	1	Ref	1	Ref	1	Ref	1	Ref	
30-34 years	0.86	0.67, 1.10	1.01	0.83, 1.23	0.92	0.80, 1.05	0.94	0.83, 1.05	0.99	0.88, 1.12	0.95	0.86, 1.06	0.97	0.82, 1.14	0.93	0.80, 1.08	
35-39 years	0.76	0.55, 1.05	1.08	0.86, 1.36	1.06	0.90, 1.24	0.99	0.86, 1.15	1.00	0.86, 1.15	0.85	0.75, 0.97	0.97	0.81, 1.17	0.94	0.80, 1.12	
≥40 years	0.88	0.55, 1.41	1.35	0.98, 1.85	0.94	0.74, 1.21	0.93	0.74, 1.17	0.87	0.69, 1.08	0.98	0.81, 1.17	1.09	0.85, 1.39	0.95	0.75, 1.20	
Birth and infancy characteristics																	
Firstborn																	0.26
Yes	1.43	1.20, 1.70	0.86	0.74, 1.00	1.23	1.12, 1.36	0.82	0.74, 0.90	1.28	1.15, 1.42	0.84	0.76, 0.94	1.06	0.88, 1.27	0.91	0.76, 1.08	
No	1	Ref	1	Ref	1	Ref	1	Ref	1	Ref	1	Ref	1	Ref	1	Ref	

Birthweight																	0.60
<2500g	1.02	0.72, 1.44	1.07	0.81, 1.41	1.04	0.88, 1.24	1.24	1.06, 1.46	1.08	0.92, 1.26	0.92	0.77, 1.10	1.06	0.84, 1.32	1.17	0.95, 1.44	
2500g-3999g	1	Ref	1	Ref	1	Ref	1	Ref	1	Ref	1	Ref	1	Ref	1	Ref	
≥4000g	1.27	0.94, 1.71	0.94	0.72, 1.23	0.96	0.79, 1.16	1.12	0.94, 1.33	1.09	0.94, 1.26	0.98	0.83, 1.15	1.08	0.86, 1.35	0.85	0.68, 1.07	
Multiple birth																	0.09
Yes	0.44	0.22, 0.87	1.04	0.72, 1.50	1.02	0.78, 1.33	0.93	0.73, 1.20	0.84	0.64, 1.11	1.30	1.06, 1.61	0.91	0.65, 1.27	1.10	0.82, 1.47	
No	1	Ref	1	Ref	1	Ref	1	Ref	1	Ref	1	Ref	1	Ref	1	Ref	
Gestational age at birth																	0.43
Born ≥1 month before due date	1.37	0.71, 2.62	0.96	0.51, 1.80	0.72	0.49, 1.07	0.96	0.69, 1.34	0.87	0.64, 1.18	1.26	0.98, 1.61	1.13	0.77, 1.66	1.34	0.95, 1.90	
Born 2-4 weeks before due date	1.22	0.70, 2.11	0.61	0.33, 1.11	1.07	0.82, 1.40	1.07	0.83, 1.38	1.09	0.89, 1.34	0.85	0.69, 1.04	0.97	0.74, 1.26	1.01	0.79, 1.28	
Not born ≥2 weeks before due date	1	Ref	1	Ref	1	Ref	1	Ref	1	Ref	1	Ref	1	Ref	1	Ref	
Ever breastfed																	0.52
Yes	0.90	0.73, 1.10	0.93	0.79, 1.10	1.01	0.91, 1.11	0.93	0.85, 1.02	1.00	0.92, 1.10	0.91	0.83, 0.99	0.98	0.86, 1.11	1.04	0.93, 1.17	
No	1	Ref	1	Ref	1	Ref	1	Ref	1	Ref	1	Ref	1	Ref	1	Ref	
Ever fed soy formula																	0.12
Yes	0.28	0.04, 2.10	1.08	0.44, 2.65	1.01	0.67, 1.51	0.73	0.47, 1.11	0.98	0.75, 1.27	1.16	0.93, 1.45	1.34	1.03, 1.74	1.15	0.89, 1.48	
No	1	Ref	1	Ref	1	Ref	1	Ref	1	Ref	1	Ref	1	Ref	1	Ref	

[a]Adjusted for race/ethnicity and childhood family income

[b]Referent group is thelarche at age 11-13 years

[c]P for heterogeneity calculated from a likelihood ratio test of nested models

[d]Birth cohort 1928-1939 was excluded from DES analysis due to low prevalence of exposure.
