# Supplementary material for: Early-life exposures and age at thelarche in the Sister Study cohort
Source: Breast Cancer Res. 2021 Dec 11;23:111. doi: 10.1186/s13058-021-01490-z (PMC8666031; doi:10.1186/s13058-021-01490-z)
Supplement: Supplementary file 9 — Additional file 9: Table S6. Associations between early-life exposures and timing of thelarche in the Sister Study cohort by Bayesian family history score (BFHS) (N = 49,151) [file 13058_2021_1490_MOESM9_ESM.pdf]

Table S6. Associations between early-life exposures and timing of thelarche in the Sister Study cohort by Bayesian family history score (BFHS) (N=49,151)

	BFHS<median[a] (n=24,233)				BFHS≥median[a] (n=24,918)					
	Early (≤10 years)[b,c]		Late (≥14 years)[b,c]		Early (≤10 years)[b,c]		Late (≥14 years)[b,c]		P-het for median BFHS[d]	P-het for continuous BFHS[e]
	OR	95% CI	OR	95% CI	OR	95% CI	OR	95% CI		
Maternal pregnancy characteristics										
Diabetes									0.18	0.21
Any	1.49	1.06, 2.10	0.88	0.61, 1.28	0.95	0.65, 1.38	0.94	0.66, 1.33		
None	1	Ref	1	Ref	1	Ref	1	Ref		
Gestational hypertensive disorder									0.22	0.68
Any	1.10	0.90, 1.35	0.95	0.78, 1.15	1.40	1.17, 1.68	0.98	0.81, 1.18		
None	1	Ref	1	Ref	1	Ref	1	Ref		
DES use									0.58	0.59
Yes	1.31	1.01, 1.70	0.95	0.73, 1.23	1.17	0.94, 1.45	1.06	0.86, 1.30		
No	1	Ref	1	Ref	1	Ref	1	Ref		
Smoking during pregnancy									0.73	0.11
Yes	1.22	1.12, 1.33	1.04	0.96, 1.13	1.18	1.09, 1.27	1.01	0.94, 1.08		
No	1	Ref	1	Ref	1	Ref	1	Ref		
Farm exposure									0.16	0.40
Work and residence	1.02	0.91, 1.14	1.04	0.94, 1.15	0.99	0.87, 1.13	0.83	0.73, 0.94		
Work only	0.93	0.66, 1.32	1.20	0.91, 1.59	1.18	0.86, 1.61	1.22	0.91, 1.62		
Residence only	0.94	0.78, 1.14	0.86	0.72, 1.02	1.02	0.84, 1.24	0.87	0.72, 1.04		
None	1	Ref	1	Ref	1	Ref	1	Ref		
Age at delivery									0.17	0.84
<20 years	1.37	1.14, 1.64	0.94	0.78, 1.12	1.24	1.05, 1.46	0.92	0.78, 1.08		
20-24 years	1.01	0.91, 1.13	1.01	0.91, 1.11	1.13	1.03, 1.25	0.94	0.86, 1.02		
25-29 years	1	Ref	1	Ref	1	Ref	1	Ref		
30-34 years	0.91	0.82, 1.01	0.98	0.89, 1.07	1.00	0.90, 1.11	0.94	0.85, 1.03		
35-39 years	0.99	0.88, 1.12	1.01	0.91, 1.13	0.98	0.86, 1.12	0.88	0.78, 0.99		
≥40 years	0.83	0.69, 0.99	1.01	0.87, 1.18	1.14	0.94, 1.39	1.03	0.86, 1.22		
Birth and infancy characteristics										
Firstborn									0.26	0.81
Yes	1.23	1.12, 1.35	0.79	0.72, 0.86	1.24	1.14, 1.35	0.87	0.80, 0.95		

No	1	Ref	1	Ref	1	Ref	1	Ref		
Birthweight									0.79	0.36
<2500g	1.07	0.93, 1.24	1.19	1.05, 1.36	1.05	0.91, 1.20	1.10	0.97, 1.25		
2500g-3999g	1	Ref	1	Ref	1	Ref	1	Ref		
≥4000g	1.02	0.88, 1.19	1.05	0.91, 1.20	0.99	0.85, 1.15	0.94	0.82, 1.08		
Multiple birth									0.12	0.11
Yes	0.79	0.62, 1.00	0.98	0.81, 1.18	0.96	0.77, 1.19	1.26	1.05, 1.51		
No	1	Ref	1	Ref	1	Ref	1	Ref		
Gestational age at birth									0.10	0.03
Born ≥1 month before due date	0.91	0.68, 1.21	1.38	1.10, 1.74	0.92	0.71, 1.19	0.98	0.77, 1.24		
Born 2-4 weeks before due date	1.09	0.88, 1.34	0.81	0.66, 1.01	1.04	0.87, 1.24	1.01	0.86, 1.19		
Not born ≥2 weeks before due date	1	Ref	1	Ref	1	Ref	1	Ref		
Ever breastfed									0.78	0.97
Yes	0.99	0.91, 1.08	0.93	0.86, 1.00	0.98	0.91, 1.06	0.96	0.89, 1.03		
No	1	Ref	1	Ref	1	Ref	1	Ref		
Ever fed soy formula									0.76	0.60
Yes	1.03	0.78, 1.35	1.00	0.78, 1.30	1.13	0.92, 1.39	1.11	0.91, 1.34		
No	1	Ref	1	Ref	1	Ref	1	Ref		

[a]Median BFHS in analytic sample is 0.306425. Median BFHS is weighted to control for length-biased sampling in calculation of BFHS. N=11 women are excluded due to missing weight.

[b]Adjusted for birth cohort, race/ethnicity and childhood family income

[c]Referent group is thelarche at age 11-13 years

[d]P for heterogeneity calculated from a likelihood ratio test of nested models

[e]Model to generate p-het for continuous BFHS was weighted to control for length-biased sampling in calculation of BFHS. N=11 women are excluded due to missing weight. P-value for heterogeneity calculated from a joint Wald test of exposure*BFHS interaction terms.
