# Supplementary material for: Early-life exposures and age at thelarche in the Sister Study cohort
Source: Breast Cancer Res. 2021 Dec 11;23:111. doi: 10.1186/s13058-021-01490-z (PMC8666031; doi:10.1186/s13058-021-01490-z)
Supplement: Supplementary file 10 — Additional file 10: Table S7. Associations between early-life exposures and age at thelarche using alternate characterizations of age at thelarche in the Sister Study cohort (N = 49,162) [file 13058_2021_1490_MOESM10_ESM.pdf]

Table S7. Associations between early-life exposures and age at thelarche using alternate characterizations of age at thelarche in the Sister Study cohort (N=49,162)

	Age at thelarche[a,b]												Continuous age at thelarche (years)[b,c]	
	Earlier development						Later development							
	≤9		10		11		13		14		≥15			
	OR	95% CI	OR	95% CI	OR	95% CI	OR	95% CI	OR	95% CI	OR	95% CI	β	95% CI
Maternal pregnancy characteristics														
Diabetes														
Any	1.40	0.92, 2.12	1.04	0.75, 1.44	1.04	0.79, 1.37	0.83	0.63, 1.09	1.01	0.73, 1.40	0.69	0.45, 1.05	-0.19	-0.33, -0.04
Gestational diabetes	*0.65*	*0.28, 1.49*	*0.77*	*0.46, 1.28*	*1.06*	*0.73, 1.53*	*0.72*	*0.48, 1.07*	*1.05*	*0.67, 1.65*	*0.48*	*0.24, 0.96*	*-0.10*	*-0.31, 0.11*
Pre-pregnancy diabetes	*2.27*	*1.37, 3.75*	*1.36*	*0.88, 2.11*	*0.94*	*0.62, 1.42*	*0.88*	*0.59, 1.30*	*0.82*	*0.49, 1.39*	*0.85*	*0.48, 1.49*	*-0.35*	*-0.56, -0.14*
None	1	Ref	1	Ref	1	Ref	1	Ref	1	Ref	1	Ref	0	Ref
Gestational hypertensive disorder														
Any	1.44	1.13, 1.83	1.28	1.08, 1.51	1.23	1.07, 1.42	0.99	0.86, 1.15	0.93	0.77, 1.13	1.12	0.92, 1.37	-0.13	-0.21, -0.05
Pre-eclampsia	*1.64*	*1.19, 2.27*	*1.36*	*1.08, 1.71*	*1.21*	*0.99, 1.48*	*1.11*	*0.92, 1.35*	*1.07*	*0.83, 1.38*	*1.09*	*0.83, 1.45*	*-0.13*	*-0.23, -0.02*
Gestational hypertension	*1.07*	*0.71, 1.61*	*1.16*	*0.88, 1.52*	*1.20*	*0.96, 1.51*	*0.86*	*0.68, 1.09*	*0.70*	*0.50, 0.97*	*0.95*	*0.69, 1.32*	*-0.18*	*-0.30, -0.05*
None	1	Ref	1	Ref	1	Ref	1	Ref	1	Ref	1	Ref	0	Ref
DES use														
Yes	1.05	0.75, 1.47	1.28	1.05, 1.56	0.91	0.76, 1.10	1.06	0.89, 1.25	0.96	0.77, 1.20	1.08	0.85, 1.37	-0.02	-0.11, 0.07
No	1	Ref	1	Ref	1	Ref	1	Ref	1	Ref	1	Ref	0	Ref
Smoking during pregnancy														
Yes	1.21	1.08, 1.35	1.20	1.12, 1.29	1.06	1.00, 1.13	0.96	0.91, 1.01	0.98	0.91, 1.05	1.09	1.00, 1.18	-0.06	-0.09, -0.03
No	1	Ref	1	Ref	1	Ref	1	Ref	1	Ref	1	Ref	0	Ref
Farm exposure														
Work and residence	1.12	0.95, 1.31	0.99	0.89, 1.10	1.01	0.92, 1.10	1.07	0.98, 1.16	0.99	0.89, 1.10	0.93	0.82, 1.05	-0.02	-0.07, 0.03
Work only	0.91	0.56, 1.46	1.23	0.93, 1.63	1.12	0.88, 1.43	1.17	0.93, 1.47	1.32	1.00, 1.75	1.28	0.94, 1.75	0.09	-0.04, 0.21
Residence only	1.19	0.94, 1.51	0.89	0.75, 1.05	0.96	0.84, 1.09	0.99	0.87, 1.12	0.85	0.71, 1.00	0.85	0.70, 1.03	-0.07	-0.14, 0.01
None	1	Ref	1	Ref	1	Ref	1	Ref	1	Ref	1	Ref	0	Ref
Age at delivery														
<20 years	1.38	1.10, 1.72	1.29	1.11, 1.51	1.09	0.96, 1.25	0.95	0.84, 1.08	0.90	0.76, 1.06	0.99	0.82, 1.18	-0.15	-0.22, -0.08
20-24 years	1.13	0.98, 1.30	1.05	0.96, 1.15	0.98	0.91, 1.06	0.96	0.90, 1.03	0.91	0.83, 0.99	1.02	0.92, 1.13	-0.04	-0.08, 0.00
25-29 years	1	Ref	1	Ref	1	Ref	1	Ref	1	Ref	1	Ref	0	Ref
30-34 years	0.94	0.81, 1.09	0.97	0.89, 1.06	1.01	0.94, 1.08	1.04	0.97, 1.12	0.98	0.89, 1.07	0.94	0.85, 1.05	0.00	-0.04, 0.04

35-39 years	0.97	0.82, 1.14	0.96	0.87, 1.07	0.97	0.89, 1.06	0.95	0.88, 1.03	0.92	0.83, 1.02	0.92	0.82, 1.04	-0.03	-0.07, 0.02
≥40 years	1.06	0.84, 1.35	0.91	0.77, 1.07	0.96	0.84, 1.09	1.04	0.92, 1.17	1.02	0.87, 1.19	0.97	0.82, 1.16	0.02	-0.05, 0.09
Birth and infancy characteristics														
Firstborn														
Yes	1.39	1.24, 1.57	1.20	1.11, 1.30	1.13	1.06, 1.20	0.90	0.84, 0.95	0.84	0.77, 0.91	0.83	0.76, 0.91	-0.20	-0.23, -0.16
No	1	Ref	1	Ref	1	Ref	1	Ref	1	Ref	1	Ref	0	Ref
Birthweight														
<2500g	1.20	1.00, 1.43	1.00	0.88, 1.13	0.98	0.88, 1.08	0.99	0.90, 1.10	1.05	0.93, 1.19	1.25	1.10, 1.43	0.05	0.00, 0.11
2500g-3999g	1	Ref	1	Ref	1	Ref	1	Ref	1	Ref	1	Ref	0	Ref
≥4000g	1.16	0.96, 1.41	0.96	0.84, 1.10	1.01	0.91, 1.12	1.03	0.93, 1.13	0.95	0.83, 1.08	1.08	0.93, 1.25	0.00	-0.06, 0.06
Multiple birth														
Yes	0.62	0.44, 0.88	0.91	0.75, 1.10	0.93	0.80, 1.08	0.92	0.79, 1.06	1.06	0.88, 1.26	1.08	0.88, 1.31	0.10	0.02, 0.18
No	1	Ref	1	Ref	1	Ref	1	Ref	1	Ref	1	Ref	0	Ref
Gestational age at birth														
Born ≥1 month before due date	0.96	0.68, 1.38	0.92	0.72, 1.17	0.99	0.81, 1.20	1.09	0.91, 1.30	1.06	0.84, 1.34	1.36	1.07, 1.74	0.14	0.04, 0.24
Born 2-4 weeks before due date	0.87	0.65, 1.14	1.16	0.99, 1.37	0.97	0.84, 1.11	1.11	0.97, 1.26	0.90	0.75, 1.08	1.01	0.83, 1.24	0.00	-0.08, 0.07
Not born ≥2 weeks before due date	1	Ref	1	Ref	1	Ref	1	Ref	1	Ref	1	Ref	0	Ref
Ever breastfed														
Yes	0.95	0.85, 1.06	1.01	0.94, 1.09	1.03	0.97, 1.09	1.02	0.96, 1.08	0.95	0.88, 1.02	0.97	0.90, 1.05	-0.02	-0.05, 0.01
No	1	Ref	1	Ref	1	Ref	1	Ref	1	Ref	1	Ref	0	Ref
Ever fed soy formula														
Yes	1.32	0.98, 1.78	1.15	0.93, 1.42	1.05	0.88, 1.26	1.26	1.07, 1.48	1.06	0.85, 1.32	1.31	1.04, 1.64	0.03	-0.06, 0.12
No	1	Ref	1	Ref	1	Ref	1	Ref	1	Ref	1	Ref	0	Ref

[a]Polytomous logistic regression model with referent group of thelarche at 12 years of age

[b]Adjusted for birth cohort, race/ethnicity and childhood family income

[c]Linear regression model with continuous age at thelarche as the outcome. β coefficient represents the difference in years in mean age at thelarche between exposed and reference group
