# Supplementary material for: Early-life exposures and age at thelarche in the Sister Study cohort
Source: Breast Cancer Res. 2021 Dec 11;23:111. doi: 10.1186/s13058-021-01490-z (PMC8666031; doi:10.1186/s13058-021-01490-z)
Supplement: Supplementary file 11 — Additional file 11: Table S8. Associations between early-life exposures and timing of thelarche in the Sister Study cohort with multiple imputation of missing early-life exposure data (N = 49,162) [file 13058_2021_1490_MOESM11_ESM.pdf]

Table S8. Associations between early-life exposures and timing of thelarche in the Sister Study cohort with multiple imputation of missing early-life exposure data (N=49,162)[a]

		Model 1				Model 2				Model 3			
		Early thelarche (≤10 years)[b,c]		Late thelarche (≥14 years)[b,c]		Early thelarche (≤10 years)[b,d]		Late thelarche (≥14 years)[b,d]		Early thelarche (≤10 years)[b]		Late thelarche (≥14 years)[b]	
	Percent Imputed	OR	95% CI	OR	95% CI	OR	95% CI	OR	95% CI	OR	95% CI	OR	95% CI
Maternal pregnancy characteristics													
Diabetes	10.4												
Any		1.21	0.94, 1.55	0.90	0.70, 1.15	1.23	0.96, 1.58	0.90	0.70, 1.15				
None		1	Ref	1	Ref	1	Ref	1	Ref				
Gestational hypertensive disorder[e]	24.7												
Any		1.29	1.13, 1.47	0.97	0.84, 1.11	1.29	1.13, 1.47	0.98	0.85, 1.12	1.29	1.13, 1.48	0.98	0.85, 1.13
None		1	Ref	1	Ref	1	Ref	1	Ref	1	Ref	1	Ref
DES use[f]	15.0												
Yes		1.23	1.03, 1.45	1.03	0.87, 1.21	1.23	1.04, 1.46	1.03	0.88, 1.21	1.18	0.99, 1.40	1.03	0.88, 1.22
No		1	Ref	1	Ref	1	Ref	1	Ref	1	Ref	1	Ref
Smoking during pregnancy	4.9												
Yes		1.20	1.13, 1.27	1.02	0.97, 1.08	1.19	1.13, 1.26	1.02	0.97, 1.08				
No		1	Ref	1	Ref	1	Ref	1	Ref				
Farm exposure	3.3												
Work and residence		0.99	0.91, 1.08	0.94	0.87, 1.02	1.01	0.93, 1.10	0.93	0.86, 1.01				
Work only		1.06	0.84, 1.34	1.20	0.99, 1.47	1.07	0.84, 1.35	1.20	0.98, 1.46				
Residence only		0.98	0.86, 1.12	0.86	0.76, 0.97	0.97	0.85, 1.11	0.86	0.76, 0.97				
None		1	Ref	1	Ref	1	Ref	1	Ref				
Age at delivery	1.2												
<20 years		1.30	1.15, 1.47	0.93	0.82, 1.04	1.18	1.03, 1.34	1.05	0.92, 1.19				
20-24 years		1.08	1.01, 1.17	0.97	0.91, 1.03	1.04	0.96, 1.12	1.02	0.95, 1.09				
25-29 years		1	Ref	1	Ref	1	Ref	1	Ref				
30-34 years		0.95	0.88, 1.02	0.95	0.89, 1.01	0.97	0.90, 1.05	0.92	0.86, 0.99				
35-39 years		0.99	0.90, 1.08	0.94	0.87, 1.02	1.02	0.93, 1.11	0.91	0.84, 0.98				
≥40 years		0.95	0.84, 1.09	1.00	0.89, 1.12	0.98	0.86, 1.23	0.96	0.86, 1.08				
Birth and infancy characteristics													
Firstborn	0.5												
Yes		1.25	1.17, 1.33	0.84	0.79, 0.89	1.20	1.11, 1.28	0.80	0.75, 0.86				

No		1	Ref	1	Ref	1	Ref	1	Ref				
Birthweight[g]	25.6												
<2500g		1.06	0.96, 1.17	1.12	1.03, 1.23	1.05	0.95, 1.16	1.13	1.03, 1.24	1.08	0.95, 1.24	1.07	0.95, 1.21
2500g-3999g		1	Ref	1	Ref	1	Ref	1	Ref	1	Ref	1	Ref
≥4000g		1.00	0.90, 1.11	0.99	0.90, 1.09	1.02	0.92, 1.14	0.98	0.89, 1.08	1.03	0.92, 1.15	0.99	0.89, 1.09
Multiple birth	<0.1												
Yes		0.87	0.74, 1.02	1.11	0.98, 1.27	0.83	0.70, 0.99	1.07	0.92, 1.23				
No		1	Ref	1	Ref	1	Ref	1	Ref				
Gestational age at birth[h]	53.6												
Born ≥1 month before due date		0.93	0.77, 1.12	1.24	1.05, 1.46	0.92	0.76, 1.11	1.24	1.05, 1.47	0.90	0.74 , 1.09	1.22	1.03, 1.45
Born 2-4 weeks before due date		1.07	0.94, 1.22	0.95	0.83, 1.09	1.07	0.94, 1.21	0.96	0.83, 1.10	1.05	0.92, 1.20	0.95	0.82, 1.10
Not born ≥2 weeks before due date		1	Ref	1	Ref	1	Ref	1	Ref	1	Ref	1	Ref
Ever breastfed[i]	7.5												
Yes		0.99	0.93, 1.04	0.94	0.90, 0.99	0.98	0.93, 1.04	0.95	0.90, 1.00	0.98	0.93, 1.04	0.96	0.91, 1.01
No		1	Ref	1	Ref	1	Ref	1	Ref	1	Ref	1	Ref
Ever fed soy formula[i]	17.1												
Yes		1.10	0.93, 1.30	1.06	0.90, 1.24	1.09	0.93, 1.29	1.06	0.90, 1.24	1.09	0.93, 1.29	1.05	0.90, 1.24
No		1	Ref	1	Ref	1	Ref	1	Ref	1	Ref	1	Ref

[a]Multiple imputation using chained equations was used to generate 50 imputed datasets in PROC MI (SAS 9.4). All except for 3 participants who withdrew their data from the study were included in the imputation models, which included the outcome, all early-life exposures, all considered covariates, age at menarche and whether mom was alive at baseline. PROC MIANALYZE (SAS 9.4) was used to combine regression coefficients across 50 imputation datasets for each early-life exposure. Multiple imputation analyses presented here are limited to the 49,162 eligible women as defined for this analysis (see Figure S1).

[b]Referent group is thelarche at 11-13 years

[c]Model 1 is adjusted for birth cohort, race/ethnicity and childhood family income

[d]Model 2 is adjusted for all variable in Model 1 plus maternal age and firstborn

[e]Model 3 includes all variables in Model 2 plus any diabetes, smoking during pregnancy and multiple birth

[f]Model 3 includes all variables in Model 2 plus any diabetes, any hypertension, smoking during pregnancy and multiple birth as potential indicators of a high-risk pregnancy

[g]Model 3 includes all variables in Model 2 plus any diabetes, any hypertension, DES use, smoking during pregnancy, multiple birth and gestational age at birth

[h]Model 3 includes all variables in Model 2 plus any diabetes, any hypertension, DES use, smoking during pregnancy, and multiple birth

[i]Model 3 includes all variables in Model 2 plus multiple birth, gestational age at birth and birthweight
