# Supplementary material for: Early-life exposures and age at thelarche in the Sister Study cohort
Source: Breast Cancer Res. 2021 Dec 11;23:111. doi: 10.1186/s13058-021-01490-z (PMC8666031; doi:10.1186/s13058-021-01490-z)
Supplement: Supplementary file 12 — Additional file 12: Table S9. Associations between early-life exposures and timing of menarche in the Sister Study cohort (N = 49,130) [file 13058_2021_1490_MOESM12_ESM.pdf]

Table S9. Associations between early-life exposures and timing of menarche in the Sister Study cohort (N=49,130)[a]

	Early menarche (≤11 years)[b,c]		Late menarche (≥14 years)[b,c]	
	OR	95% CI	OR	95% CI
Maternal pregnancy characteristics				
Diabetes				
Any	1.35	1.08, 1.69	1.14	0.91, 1.43
Gestational diabetes	*1.09*	*0.77, 1.53*	*0.93*	*0.66, 1.29*
Pre-pregnancy diabetes	*1.66*	*1.21, 2.28*	*1.42*	*1.03, 1.96*
None	1	Ref	1	Ref
Gestational hypertensive disorder				
Any	1.27	1.13, 1.44	0.96	0.85, 1.09
Pre-eclampsia	*1.18*	*1.00, 1.39*	*0.98*	*0.83, 1.16*
Gestational hypertension	*1.31*	*1.08, 1.58*	*0.89*	*0.73, 1.09*
None	1	Ref	1	Ref
DES use				
Yes	1.21	1.05, 1.41	0.98	0.85, 1.14
No	1	Ref	1	Ref
Smoking during pregnancy				
Yes	1.14	1.08, 1.19	1.01	0.97, 1.06
No	1	Ref	1	Ref
Farm exposure				
Work and residence	1.01	0.94, 1.09	1	0.93, 1.08
Work only	0.89	0.72, 1.10	1.13	0.94, 1.36
Residence only	0.99	0.88, 1.11	1.06	0.95, 1.18
None	1	Ref	1	Ref
Age at delivery				
<20 years	1.24	1.11, 1.38	0.90	0.81, 1.01
20-24 years	1.05	0.98, 1.12	1.02	0.96, 1.09
25-29 years	1	Ref	1	Ref
30-34 years	1.03	0.96, 1.10	0.98	0.93, 1.04
35-39 years	1.02	0.94, 1.10	0.89	0.83, 0.96
≥40 years	1.05	0.94, 1.17	0.95	0.85, 1.06
Birth and infancy characteristics				
Firstborn				
Yes	1.14	1.08, 1.20	0.90	0.85, 0.95
No	1	Ref	1	Ref
Birthweight				
<2500g	1.15	1.05, 1.25	1.05	0.96, 1.14
2500g-3999g	1	Ref	1	Ref

≥4000g	1.08	0.99, 1.19	1.11	1.01, 1.21
Multiple birth				
Yes	0.92	0.80, 1.05	1.00	0.89, 1.14
No	1	Ref	1	Ref
Gestational age at birth				
Born ≥1 month before due date	1.08	0.92, 1.27	1.07	0.92, 1.26
Born 2-4 weeks before due date	1.00	0.89, 1.13	0.86	0.77, 0.97
Not born ≥2 weeks before due date	1	Ref	1	Ref
Ever breastfed				
Yes	0.97	0.92, 1.02	1.00	0.95, 1.05
No	1	Ref	1	Ref
Ever fed soy formula				
Yes	1.05	0.90, 1.23	1.19	1.04, 1.37
No	1	Ref	1	Ref

[a]32 women missing age at menarche were excluded from this analysis. N=10,055 women with early menarche, 27,613 women with average menarche and 11,462 women with late menarche.

[b]Adjusted for birth cohort, race/ethnicity and childhood family income

[c]Referent group is menarche at 12-13 years
