# Supplementary material for: Early-life exposures and age at thelarche in the Sister Study cohort
Source: Breast Cancer Res. 2021 Dec 11;23:111. doi: 10.1186/s13058-021-01490-z (PMC8666031; doi:10.1186/s13058-021-01490-z)
Supplement: Supplementary file 13 — Additional file 13: Table S10. Associations between early-life exposures and early thelarche (≤ 10 years) and/or early menarche (≤ 11 years) in the Sister Study cohort (N = 49,130) [file 13058_2021_1490_MOESM13_ESM.pdf]

Table S10. Associations between early-life exposures and early thelarche (≤10 years) and/or early menarche (≤11 years) in the Sister Study cohort (N=49,130)[a]

	Early menarche without early thelarche (n=5821)[b,c]		Early thelarche without early menarche (n=2370)[b,c]		Early thelarche and early menarche (n=4234)[b,c]	
	OR	95% CI	OR	95% CI	OR	95% CI
Maternal pregnancy characteristics						
Diabetes						
Any	1.21	0.92, 1.60	1.00	0.65, 1.53	1.41	1.05, 1.89
Gestational diabetes	*1.17*	*0.78, 1.75*	*0.61*	*0.28, 1.30*	*0.98*	*0.60, 1.59*
Pre-pregnancy diabetes	*1.20*	*0.81, 1.80*	*1.55*	*0.92, 2.62*	*1.97*	*1.36, 2.86*
None	1	Ref	1	Ref	1	Ref
Gestational hypertensive disorder						
Any	1.20	1.04, 1.39	1.08	0.87, 1.35	1.42	1.22, 1.66
Pre-eclampsia	*1.03*	*0.83, 1.28*	*1.15*	*0.86, 1.55*	*1.42*	*1.15, 1.76*
Gestational hypertension	*1.29*	*1.02, 1.63*	*0.91*	*0.62, 1.34*	*1.41*	*1.09, 1.81*
None	1	Ref	1	Ref	1	Ref
DES use						
Yes	1.08	0.89, 1.30	0.94	0.71, 1.26	1.40	1.16, 1.69
No	1	Ref	1	Ref	1	Ref
Smoking during pregnancy						
Yes	1.09	1.03, 1.16	1.19	1.09, 1.30	1.22	1.14, 1.30
No	1	Ref	1	Ref	1	Ref
Farm exposure						
Work and residence	1.02	0.93, 1.11	1.03	0.90, 1.19	1.00	0.91, 1.11
Work only	0.86	0.66, 1.11	1.18	0.84, 1.65	0.87	0.65, 1.17
Residence only	0.95	0.83, 1.10	1.02	0.82, 1.26	1.00	0.85, 1.17
None	1	Ref	1	Ref	1	Ref
Age at delivery						
<20 years	1.26	1.11, 1.44	1.39	1.15, 1.68	1.36	1.18, 1.58
20-24 years	1.05	0.97, 1.13	1.18	1.06, 1.33	1.06	0.97, 1.16
25-29 years	1	Ref	1	Ref	1	Ref
30-34 years	1.04	0.96, 1.12	0.88	0.78, 0.99	1.01	0.92, 1.10
35-39 years	1.10	1.00, 1.21	1.05	0.91, 1.21	0.99	0.89, 1.11
≥40 years	1.15	1.01, 1.31	1.00	0.82, 1.22	0.95	0.81, 1.12
Birth and infancy characteristics						
Firstborn						
Yes	1.11	1.03, 1.18	1.29	1.16, 1.42	1.32	1.23, 1.42
No	1	Ref	1	Ref	1	Ref
Birthweight						
<2500g	1.18	1.06, 1.31	1.02	0.87, 1.20	1.07	0.95, 1.21
2500g-3999g	1	Ref	1	Ref	1	Ref
≥4000g	1.08	0.97, 1.21	1.02	0.87, 1.21	1.01	0.89, 1.15
Multiple birth						
Yes	0.98	0.84, 1.15	0.92	0.72, 1.18	0.81	0.67, 0.99
No	1	Ref	1	Ref	1	Ref

Gestational age at birth						
Born ≥1 month before due date	1.08	0.89, 1.31	0.71	0.50, 1.01	0.99	0.79, 1.24
Born 2-4 weeks before due date	1.03	0.89, 1.19	1.09	0.88, 1.35	1.07	0.91, 1.27
Not born ≥2 weeks before due date	1	Ref	1	Ref	1	Ref
Ever breastfed						
Yes	0.95	0.89, 1.01	0.98	0.89, 1.07	1.00	0.93, 1.07
No	1	Ref	1	Ref	1	Ref
Ever fed soy formula						
Yes	0.94	0.78, 1.14	1.06	0.82, 1.37	1.08	0.88, 1.32
No	1	Ref	1	Ref	1	Ref

[a]32 women missing age at menarche were excluded from this analysis

[b]Adjusted for birth cohort, race/ethnicity and childhood family income

[c]Referent group is neither early thelarche nor early menarche (thelarche at age 11 or later and menarche at age 12 or later, n=36,705)
